# Draft Genome Sequence of *Micrococcus* sp. Strain MS-AsIII-49, an Arsenate-Reducing Isolate from Tropical Metal-Rich Sediment

**DOI:** 10.1128/genomeA.00122-15

**Published:** 2015-04-16

**Authors:** Patrícia S. Costa, Diogo A. Tschoeke, Bruno S. O. Silva, Fabiano Thompson, Mariana P. Reis, Edmar Chartone-Souza, Andréa M. A. Nascimento

**Affiliations:** a Departamento de Biologia Geral, Instituto de Ciências Biológicas, Universidade Federal de Minas Gerais, Belo Horizonte, Minas Gerais, Brazil; b Laboratório de Microbiologia, Universidade Federal do Rio de Janeiro, Rio de Janeiro, Brazil

## Abstract

*Micrococcus* sp. strain MS-AsIII-49, which was isolated from a tropical metal-polluted stream sediment in Brazil, has the ability to reduce AsV to AsIII. Analysis of its draft genome revealed 186 contigs with a total size of 2,440,924 bp encoding several metal resistance genes.

## GENOME ANNOUNCEMENT

Members of the *Micrococcus* genus are found in the environment as saprophyte or commensal in a wide variety of ecosystems, such as human skin, water, dust, and soil, among others (1–8). This genus consists of nine recognized species. So far, only the *M. luteus* species has been sequenced, and its whole genome revealed a small size (2,501,097 bp) with only a few adaptation genes, leading to a very strict ecological niche (9). In this context, the *Micrococcus* sp. strain MS-ASIII-49, described in the present study, was isolated from a tropical metal-rich stream sediment, affected by human settlement, especially arsenic, NO_3_^−^ and NH_4_^+^ (10). This strain was phenotypically characterized as AsV-reducing bacteria. In addition, the genome sequencing of *Micrococcus* sp. strain MS-ASIII-49 provided insight into the genomic diversity and evolution of the *Micrococcus* genus.

For the genome sequencing of *Micrococcus* sp. strain MS-AsIII-49, a mate pair library was constructed and sequenced on an Illumina Hiseq 2000 platform at the Beijing Genomics Institute. The reads were subjected to preprocessing consisting of screens for errors and quality-based trimming using the *prinseq-lite* algorithm (11). The reads obtained were assembled using *de novo* assembly tool in the MIRA 4 software. After the assembly, the contigs were automatically annotated using the Rapid Annotations using Subsystems Technology (RAST) server (12).

The size of the *Micrococcus* sp. MS-AsIII-49 draft genome is 2,440,924 bp, comprising 186 contigs with a G+C content of 73.08%. The *N*_50_ contig length was 27,810 bp, the largest contig length was 82,396 bp, and the smallest was 198 bp. In total, the numbers of putative coding sequences (CDS) was 2,189 in *Micrococcus* sp. MS-AsIII-49. Only one copy of the 5S rRNA, 16S rRNA, and 23S rRNA genes were found. Additionally, 43 tRNA sequences were found in *Micrococcus* sp. MS-AsIII-49. The subsystem analysis revealed predominance of subsystems related to cell maintenance, such as amino acids and derivatives, carbohydrates, and protein metabolism, among others. A comparison between the genome sequence of *Micrococcus* sp. MS-AsIII-49 and *M. luteus*, using the SEED comparative tool, revealed 165 genes found only in *Micrococcus* sp. MS-AsIII-49, and 55.15% (91/165) of these genes were annotated as hypothetical proteins. The identified genes were related to mobile elements as integrase and normal cell function as glycosyltransferase, acetyltransferase, and DNA-methyltransferase, among others. The presence of several genes in the virulence, disease, and defense and stress response subsystems in the *Micrococcus* sp. MS-AsIII-49 genome suggests that this strain harbors a genomic repertory able to deal with metals and other environmental toxicants, which would provide an advantage in sediment impacted by human settlement. The analysis of the virulence, disease, and defense subsystem provided insight into *Micrococcus* sp. MS-AsIII-49 metal resistance identifying specific proteins related to arsenic resistance as ArsR, ArsC, and ACR3; mercury resistance as MerR and MerA; copper resistance as CopZ, CopC, CopB, multicopper oxidase, and CtpA; and cobalt-zinc-cadmium resistance as CzcD protein.

### Nucleotide sequence accession numbers. 

This whole-genome shotgun project has been deposited at DDBJ/EMBL/GenBank under the accession no. JXSP00000000. The version described in this paper is version JXSP01000000.
